# Unraveling an Unusual Phenocopy of Hypertrophic Cardiomyopathy: MELAS Syndrome

**DOI:** 10.3390/diagnostics11020295

**Published:** 2021-02-12

**Authors:** Anna B. Reid, Luigi Venetucci, Matthias Schmitt, Gaetano Nucifora

**Affiliations:** 1 Cardiac Imaging Unit, North West Heart Centre, Manchester University NHS Foundation Trust, Manchester M23 9LT, UK; anna.reid@mft.nhs.uk (A.B.R.); matthias.schmitt@mft.nhs.uk (M.S.); 2 Manchester Heart Centre, Manchester Royal Infirmary, Manchester University NHS Foundation Trust, Manchester M13 9WL, UK; luigi.venetucci@manchester.ac.uk; 3 Institute of Cardiovascular Sciences, University of Manchester, Manchester M13 9PL, UK

**Keywords:** cardiac magnetic resonance, hypertrophic cardiomyopathy, left ventricular hypertrophy, MELAS syndrome

## Abstract

The mitochondrial myopathy, encephalopathy, lactic acidosis, and stroke-like episodes (MELAS) syndrome is an uncommon cause of cardiac hypertrophy, fibrosis, and dysfunction. It shares similar features to numerous other causes of left ventricular hypertrophy, and therefore, because of its rarity, may not be immediately considered as a diagnosis. Prompt recognition of clinical and cardiac imaging features may expedite diagnosis and management. We report the case of a 38-year-old man admitted with neurological symptoms and in whom the diagnostic workup led to the diagnosis of MELAS syndrome with cardiac involvement.

**Figure 1:**
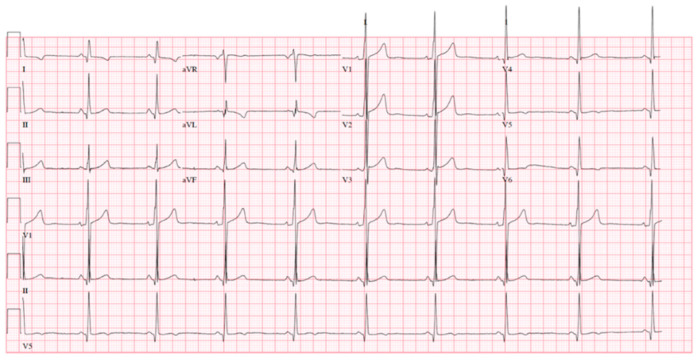
A 38-year-old male with a past history of sensorineural deafness from his late teenage years and type 1 diabetes mellitus presented to the accident and emergency department with headaches, change in personality, ataxia, and left-sided facial asymmetry. Initial computed tomography scan of the brain identified three areas of non-specific oedema in the right cerebral hemisphere. Subsequent magnetic resonance imaging (MRI) of the brain identified cortical swelling of the right parietal lobe, occipital lobe, and temporal lobe, with areas of cortical diffusion restriction, basal ganglia calcification, and diffuse cerebral atrophy. Lactic acidosis (plasma lactate 3.51 mmol/L, reference range 0.50–2.20) was identified on arterial blood gases. Electrocardiogram revealed voltage criteria for left ventricular hypertrophy (LVH) with non-specific repolarization abnormalities in the lateral leads and short PR interval (Figure 1). Subsequent echocardiography (Figure 2, Panels **A**,**B**) identified concentric symmetrical left ventricular hypertrophy with a maximal wall thickness of 1.6 cm and an ejection fraction of 40% (Supplemental Video S1). Grade II diastolic dysfunction was also evident (Figure 2, Panel **C**, E/e’ 12).

**Figure 2:**
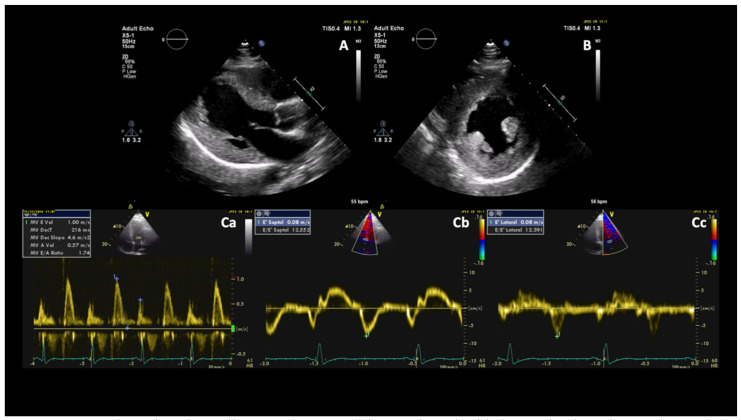
Transthoracic echocardiogram (parasternal long-axis and mid short-axis views in Panels **A**,**B**) showed concentric symmetrical LVH with a maximal wall thickness of 1.6 cm. Pulsed wave Doppler interrogation of the mitral valve at the level of the leaflet tips (Panel **Ca**) demonstrated increased E/A ratio (1.7) and E/e’ ratio (obtained after measurement of septal and lateral annulus velocities by tissue-Doppler imaging, Panels **Cb**,**Cc**) was 12, indicating grade II diastolic dysfunction. Cardiac MRI was performed on a 3T scanner (Magnetom Skyra, Siemens, Erlangen, Germany), confirming increased left ventricular mass (117 g/m^2^), an ejection fraction of 45% with reduced longitudinal function, and normal left atrial size. Native and post-contrast T1 mapping (motion-corrected modified Look-Locker inversion recovery (MOLLI) sequence) identified widespread heterogeneity in the signal, with elevated native T1 values and extracellular volume corresponding to areas of mid-wall and epicardial enhancement, particularly in the lateral wall (Figure 3A–D).

**Figure 3:**
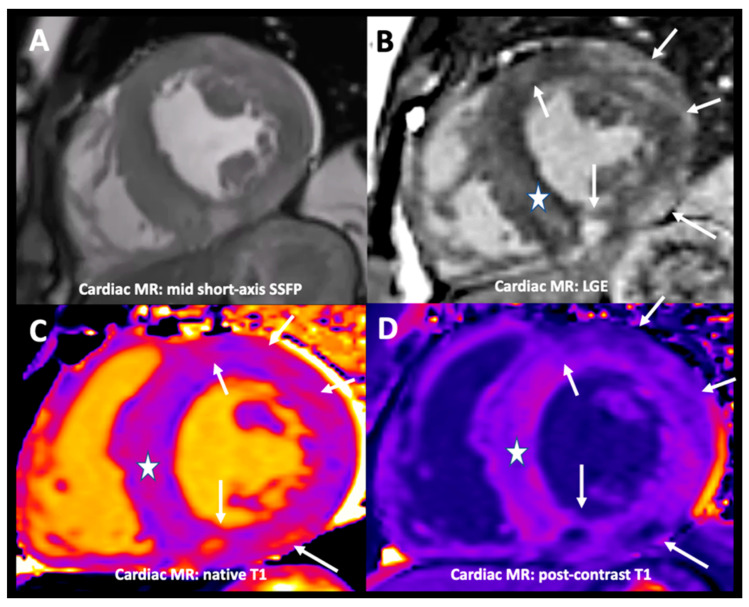
Steady-state free precession cardiac MRI (Panel **A**) confirmed a concentric increase of left ventricular wall thickness. Contrast-enhanced inversion-recovery T1-weighted fast gradient-echo images obtained 10 min after injection of gadolinium chelate contrast agent (i.e., late gadolinium enhancement (LGE) technique, Panel **B**) demonstrated areas of mid-wall and epicardial enhancement, particularly in the lateral wall, as well as hyper-enhancement at both RV insertion points (arrows) and fainter mid-wall septal hyper-enhancement (asterisk). Native and post-contrast T1 mapping (Panels **C**,**D**, respectively) identified widespread heterogeneity in the signal, with elevated native T1 values and extracellular volume corresponding to the previously described areas of myocardial hyper-enhancement (asterisk and arrows in both Panels). Clinical presentation and cardiac MRI features were deemed unusual for sarcomeric hypertrophic cardiomyopathy (HCM); genetic sequencing was performed, identifying a m.3243A > G mutation in the mitochondrial tRNA^Leu (UUR)^ (MTTL1) gene, consistent with the working diagnosis of mitochondrial encephalopathy with lactic acidosis and stroke-like episodes (MELAS) syndrome. Careful family history revealed no suggestion of affected family members; indeed, the patient’s brother to date has no manifestations of the disease. MELAS syndrome is one of several inherited conditions included in a heterogeneous group of metabolic myopathies (MMs) that result from abnormalities in mitochondrial DNA. The clinical presentation of metabolic disorders is seen in approximately 1 in 10,000 adults; however, mutations are found in 1:200 of the background population [1]. The mutation in our case is typical, accounting for eighty percent of MELAS cases. Reduced mitochondrial function impairs oxidative phosphorylation; abnormal myocardial energy handling is a known factor in the development of cardiac hypertrophy, fibrosis, and dysfunction [2]. This maternally inherited syndrome has a variable phenotypic presentation, both in terms of age of presentation and organ involvement. This heterogeneity is thought to reflect heteroplasmy, variable amounts of mutant and wild-type DNAs in different tissues, and the different energy demands of different organs. Given that the heart is the most energy-consuming organ within the human body, it is not then surprising that cardiac involvement is frequently seen in patients with MMs and in the majority of patients with MELAS. In addition to the cardiac involvement, clinical manifestations include cognitive decline, epilepsy, muscle weakness, vision and hearing impairment, diabetes mellitus, and other endocrinopathies. Indeed, at follow-up, our patient has unfortunately developed several of these manifestations. Our case represents the typical cardiac phenotype of hypertrophic cardiomyopathy. This may progress to a dilated cardiomyopathy (DCM); however, DCM as the initial presenter is less common [3]. Malignant arrhythmias, heart failure, and sudden cardiac death may occur, with atrial tachyarrhythmias and pre-excitation also being frequently reported. Despite a short PR interval and frequent complaints of palpitations, repeated Holter monitoring and subsequent implantable loop recordings have failed to identify sustained arrhythmia. Brady-arrhythmias occur less commonly and have not yet been seen in our case. The major cardiac differential diagnoses, in this case, are Anderson–Fabry disease and HCM. Certainly, Anderson–Fabry may present with stroke and other systemic features such as deafness. Morphologically, the left ventricles in these conditions may be very similar but will differ in their patterns of LGE and tissue characterization using T1 mapping (Figure 4). Multiparametric cardiac MRI may provide a rapid distinction between these cardiomyopathies, unlike genetic or metabolic testing, which may take weeks to months. The cardiac MRI findings in our case of patchy, widespread, non-ischemic type LGE are also typical for the MELAS cardiomyopathy and are thought to arise from diffuse interstitial fibrosis, myocardial disarray, and replacement fibrosis in the context of myocyte death [4]. Mitochondrial angiopathy, as seen cerebrally causing the stroke-like episodes, and myocardial inflammation may also contribute to the development of fibrosis and organ failure. In addition to its diagnostic capabilities, T1 (and T2) mapping may offer further insight into these pathological processes. This case highlights the importance of multimodality imaging in differentiating hypertrophic cardiomyopathies [5,6,7,8].

**Figure 4:**
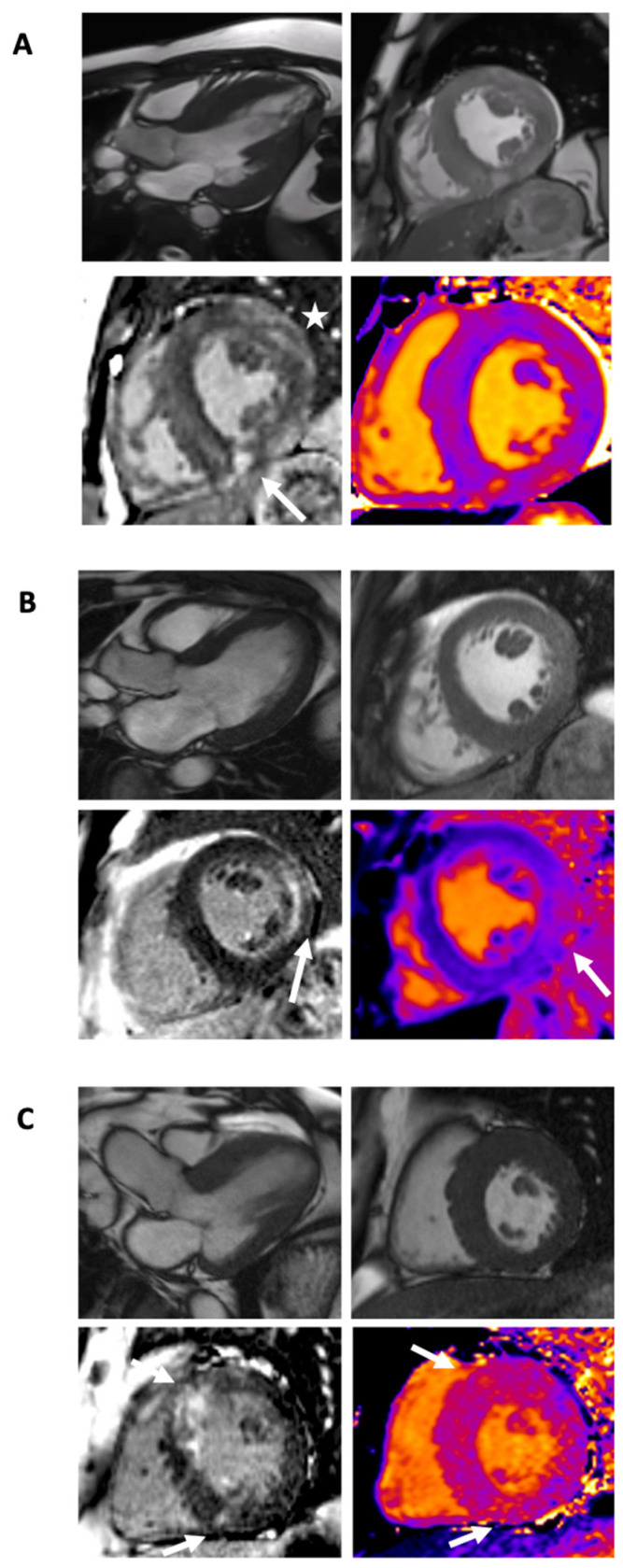
The differential diagnosis: cardiac MRI in left ventricular hypertrophy. Clockwise from left: still image from cine steady-state free precession three-chamber and mid-short-axis views, mid-short-axis T1 map, and mid-short-axis LGE image. Note how visually the three ventricles in Panels (**A**–**C**) look similar in morphology but have different tissue characteristics. (**A**) MELAS: There are moderate symmetrical left ventricular hypertrophy and normal right ventricular wall thickness. Native T1 mapping values are within normal limits with islands of higher values. LGE imaging identifies patchy mid-wall enhancement predominantly affecting the lateral wall (star), but also the septum with denser epicardial enhancement inferiorly (arrow). (**B**) Anderson–Fabry Disease: There is moderate symmetrical left ventricular hypertrophy with septal attenuation, with right ventricular hypertrophy. Native T1 mapping values are low (darker purple) in the septum, and normal in the inferolateral wall. This corresponds with LGE in the inferolateral wall (arrows). (**C**) Hypertrophic cardiomyopathy: There is asymmetrical hypertrophy affecting the basal to mid anterior wall and anteroseptum. Native T1 values are diffusely higher, with areas of relatively higher values (arrows) at both septal RV insertion points. LGE is seen in the septum and insertion points, in the area of maximal hypertrophy.

## Data Availability

Not applicable.

## Supplementary Materials

The following are available online at https://www.mdpi.com/2075-4418/11/2/295/s1, Video S1: Apical 4-chamber view on transthoracic echocardiography.
